# The Prescriber's Pharmacopœia: Containing All the Medicines in the London Pharmacopœia, Arranged in Classes According to Their Action, with Their Composition and Doses

**Published:** 1843-01

**Authors:** 


ART. IV.—*The Prescriber's Pharmacopœia: containing all the Medicines in the London Pharmacopœia, arranged in classes according to their action, with their composition and doses.* By a PRACTISING PHYSICIAN. Second Edition, corrected and enlarged.—*London*, 1843. 32mo, pp. 149.

WE gave an account of the first edition of this little work in a former Number, (vol. XII. p. 221.) Its having reached a second edition so soon, justifies the opinion we then gave of its value. In the new edition it is much improved in plan, and contains a good deal of new matter. It is decidedly one of the most useful little works that has appeared for a long time; and we recommend it to the attention of every prescriber.

